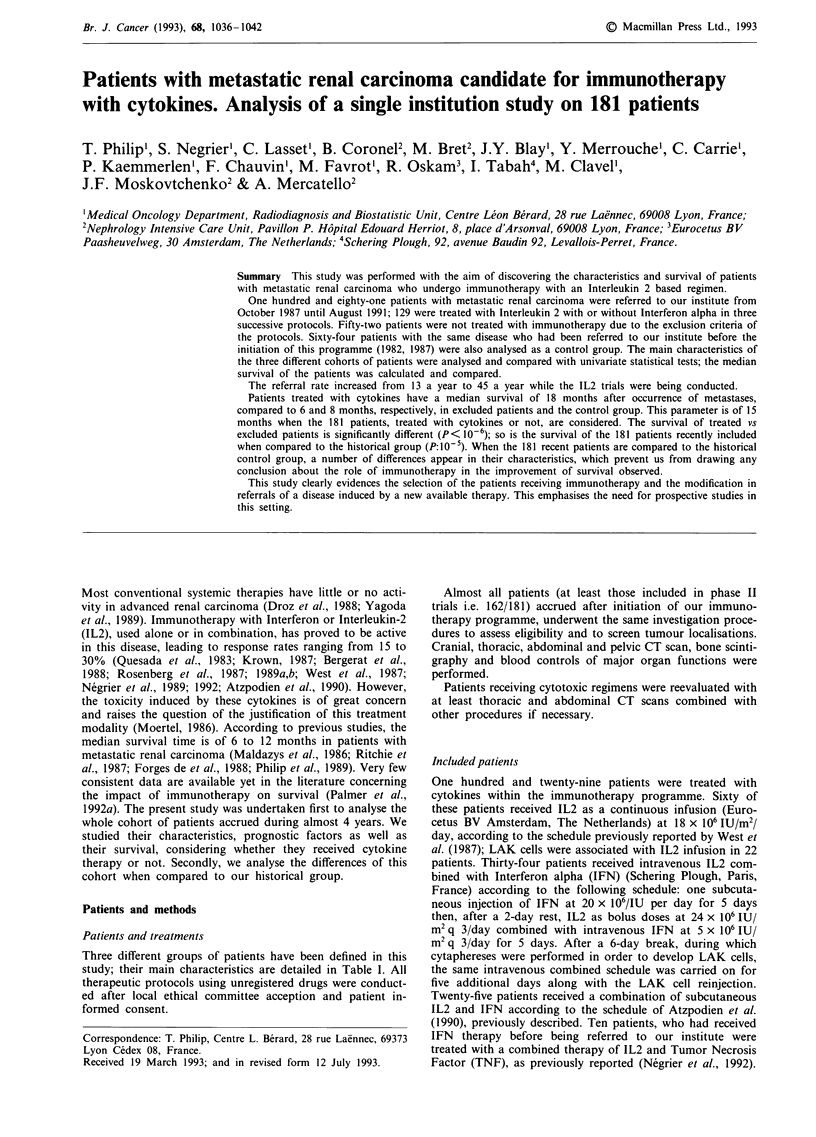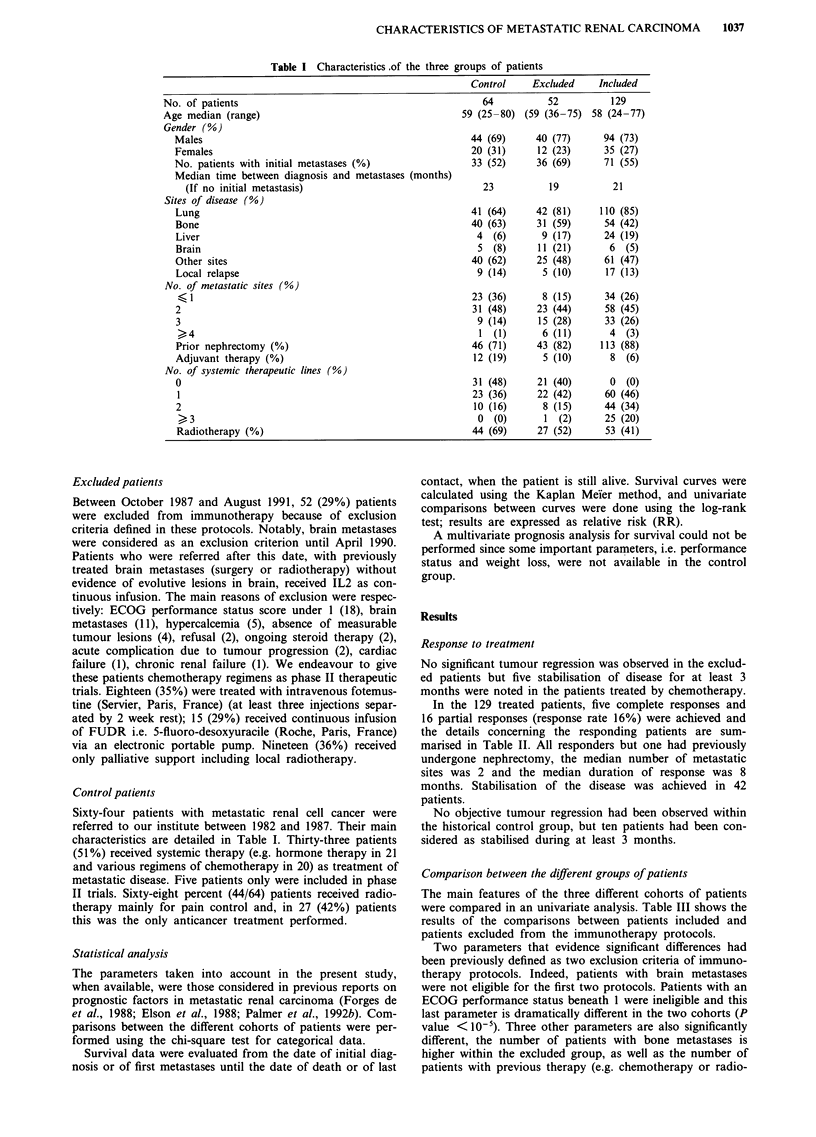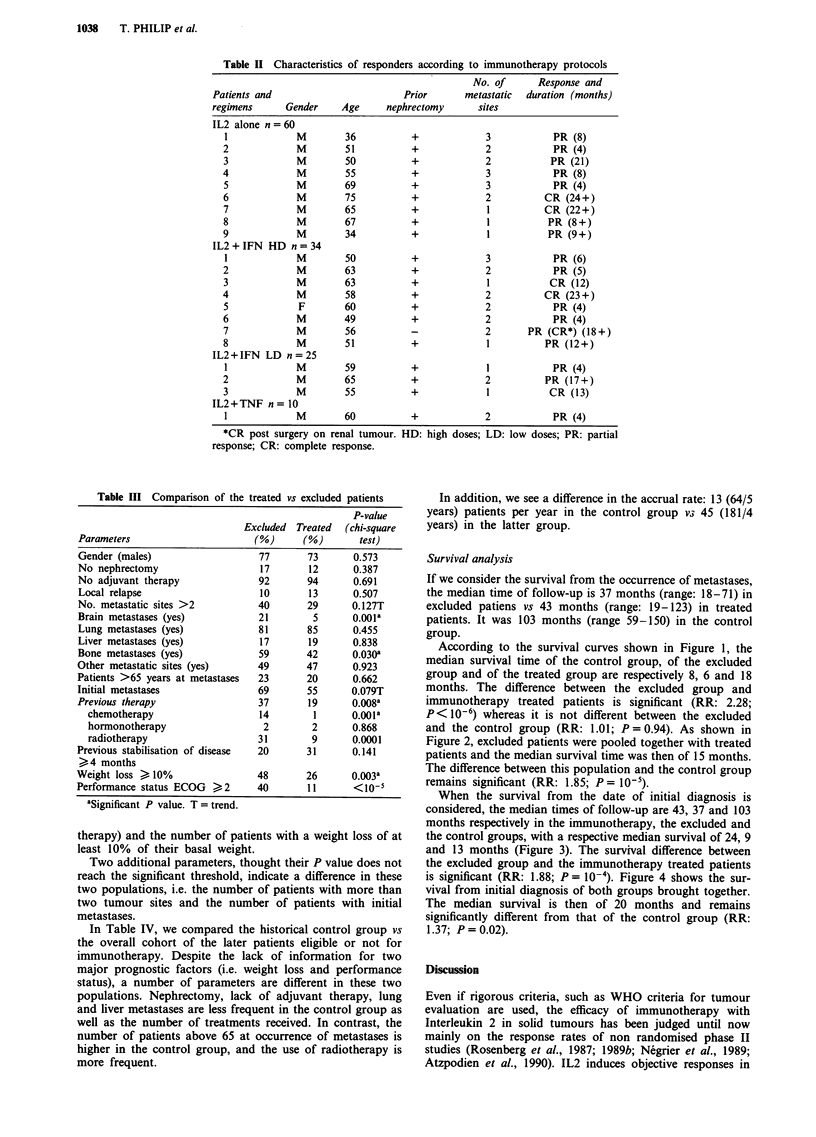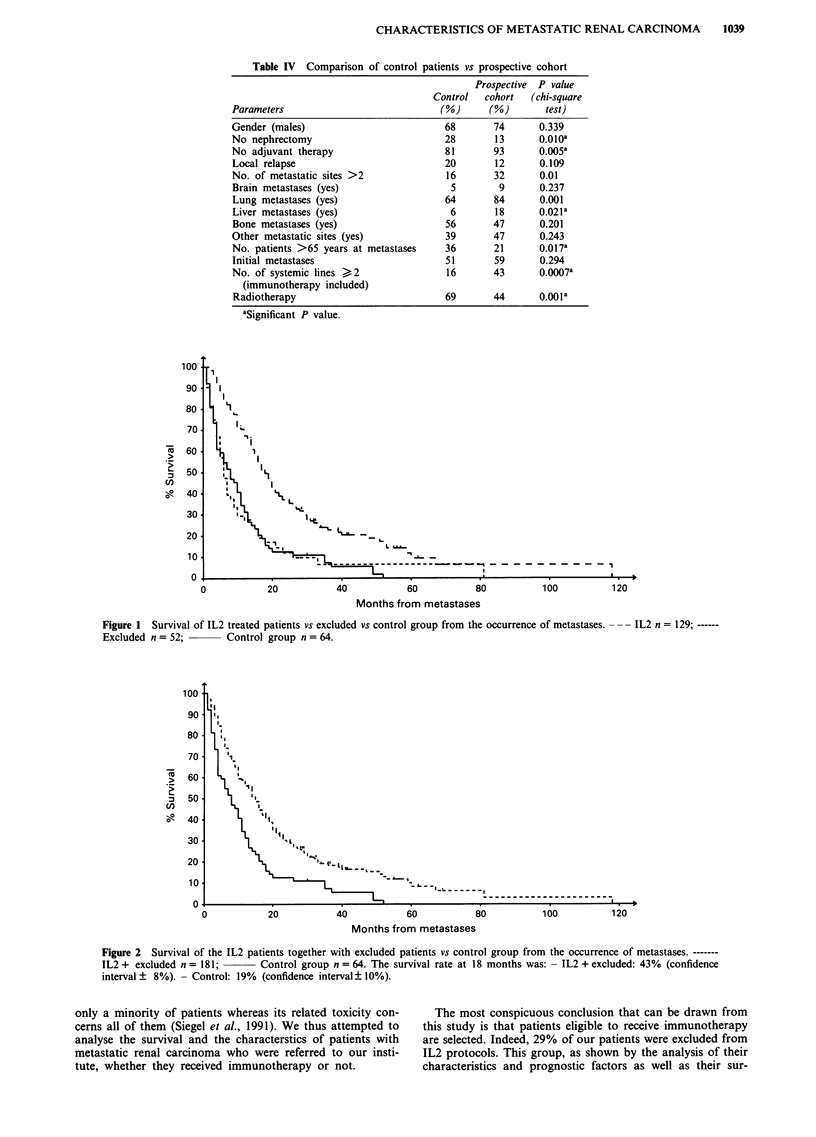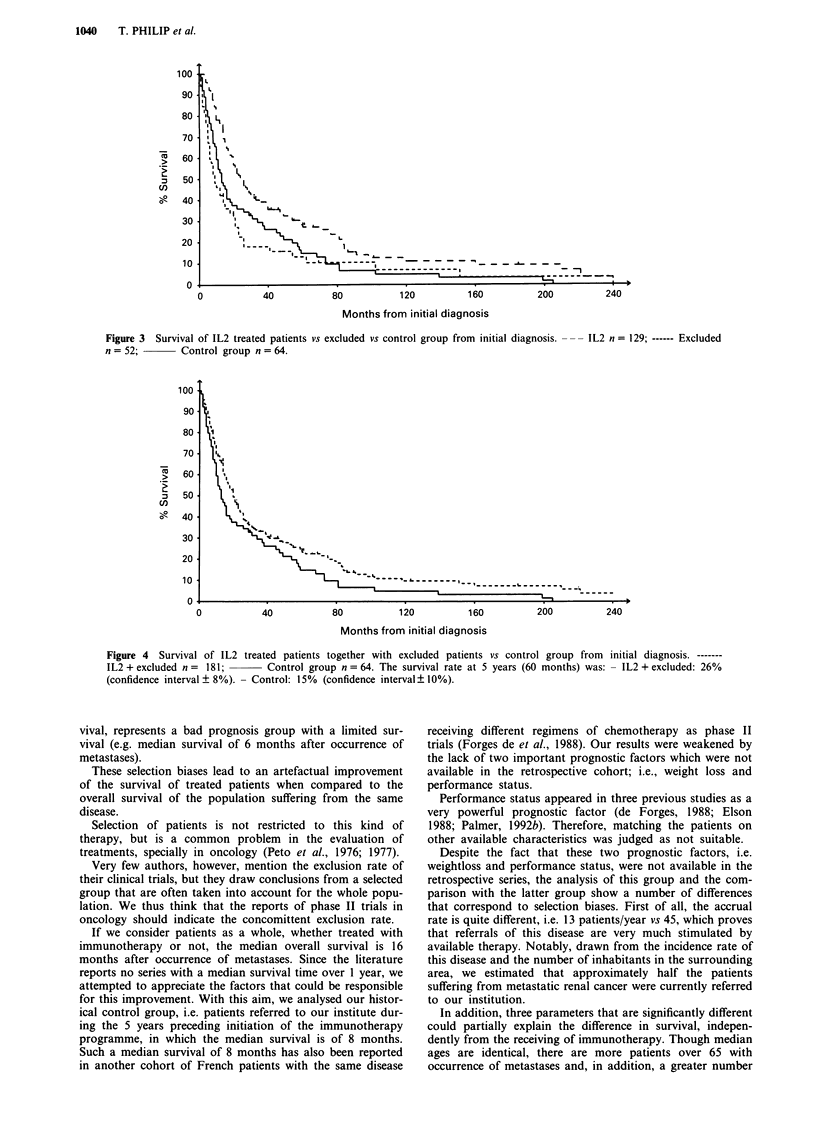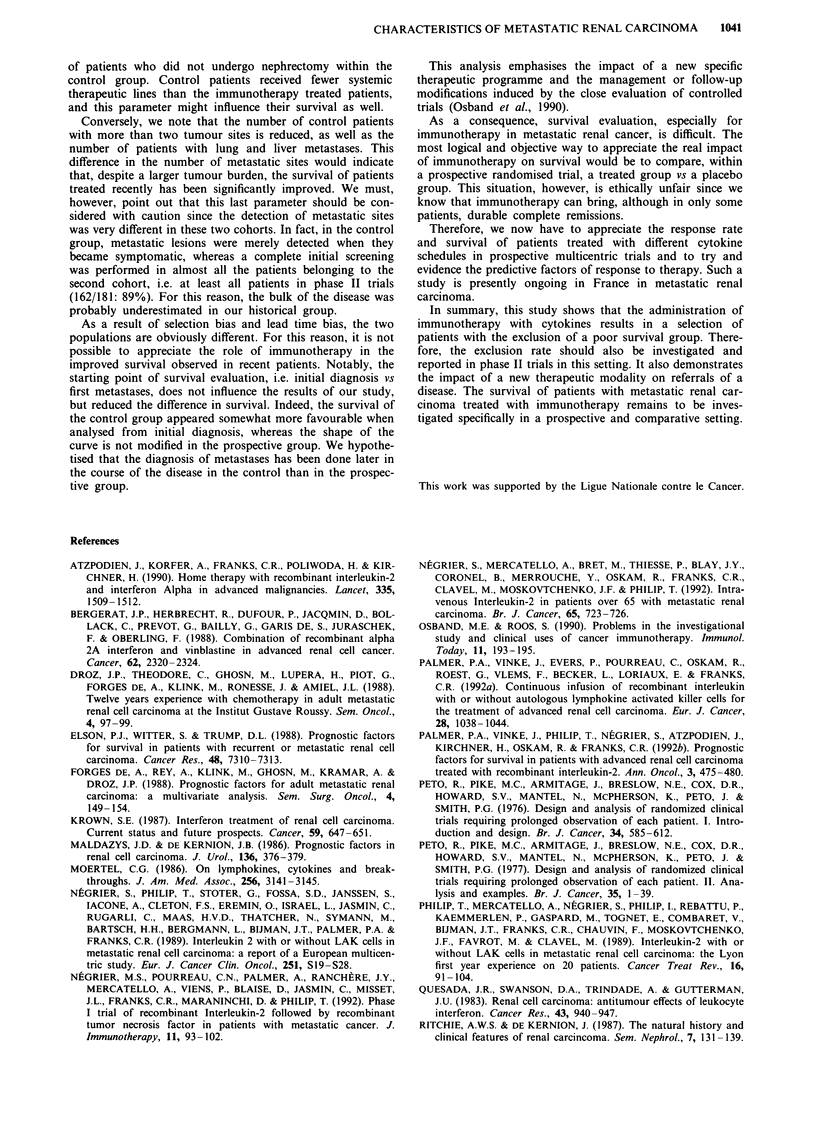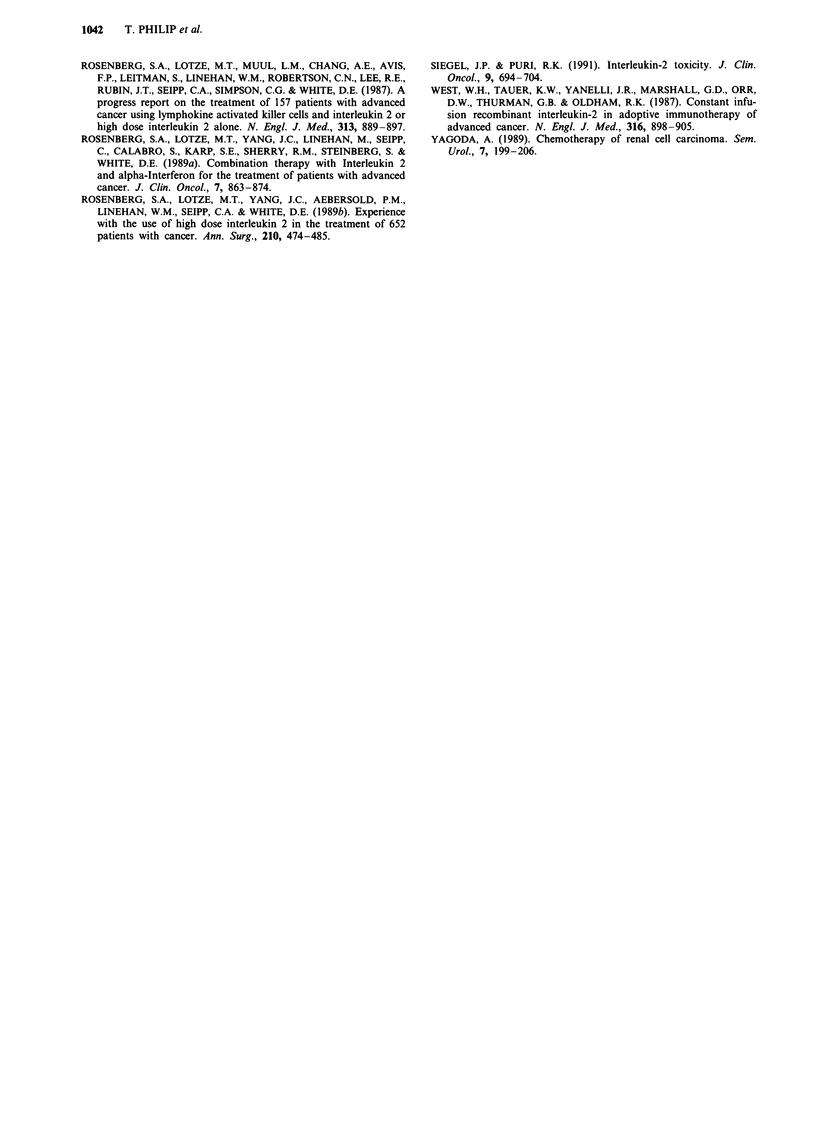# Patients with metastatic renal carcinoma candidate for immunotherapy with cytokines. Analysis of a single institution study on 181 patients.

**DOI:** 10.1038/bjc.1993.476

**Published:** 1993-11

**Authors:** T. Philip, S. Negrier, C. Lasset, B. Coronel, M. Bret, J. Y. Blay, Y. Merrouche, C. Carrie, P. Kaemmerlen, F. Chauvin

**Affiliations:** Medical Oncology Department, Centre Léon Bérard, Lyon, France.

## Abstract

This study was performed with the aim of discovering the characteristics and survival of patients with metastatic renal carcinoma who undergo immunotherapy with an Interleukin 2 based regimen. One hundred and eighty-one patients with metastatic renal carcinoma were referred to our institute from October 1987 until August 1991; 129 were treated with Interleukin 2 with or without Interferon alpha in three successive protocols. Fifty-two patients were not treated with immunotherapy due to the exclusion criteria of the protocols. Sixty-four patients with the same disease who had been referred to our institute before the initiation of this programme (1982, 1987) were also analysed as a control group. The main characteristics of the three different cohorts of patients were analysed and compared with univariate statistical tests; the median survival of the patients was calculated and compared. The referral rate increased from 13 a year to 45 a year while the IL2 trials were being conducted. Patients treated with cytokines have a median survival of 18 months after occurrence of metastases, compared to 6 and 8 months, respectively, in excluded patients and the control group. This parameter is of 15 months when the 181 patients, treated with cytokines or not, are considered. The survival of treated vs excluded patients is significantly different (P < 10(-6); so is the survival of the 181 patients recently included when compared to the historical group (P:10(-5). When the 181 recent patients are compared to the historical control group, a number of differences appear in their characteristics, which prevent us from drawing any conclusion about the role of immunotherapy in the improvement of survival observed. This study clearly evidences the selection of the patients receiving immunotherapy and the modification in referrals of a disease induced by a new available therapy. This emphasises the need for prospective studies in this setting.


Br. J. Cancer (1993), 68, 1036–1042 © Macmillan Press Ltd., 1993

### Patients with metastatic renal carcinoma candidate for immunotherapy with cytokines. Analysis of a single institution study on 181 patients

T. Philip[1], S. Negrier[1], C. Lasset[1], B. Coronel[2], M. Bret[2], J.Y. Blay[1], Y. Merrouche[1], C. Carrie[1], P. Kaemmerlen[1], F. Chauvin[1], M. Favrot[1], R. Oskam[3], I. Tabah[4], M. Clavel[1], J.F. Moskovtchenko[2] & A. Mercatello[2]

[1]*Medical Oncology Department, Radiodiagnosis and Biostatistic Unit, Centre Léon Bérard, 28 rue Laënnec, 69008 Lyon, France;* [2]*Nephrology Intensive Care Unit, Pavillon P. Hôpital Edouard Herriot, 8, place d'Arsonval, 69008 Lyon, France;* [3]*Eurocetus BV Paasheuvelweg, 30 Amsterdam, The Netherlands;* [4]*Schering Plough, 92, avenue Baudin 92, Levallois-Perret, France.*

**Summary** This study was performed with the aim of discovering the characteristics and survival of patients with metastatic renal carcinoma who undergo immunotherapy with an Interleukin 2 based regimen.

One hundred and eighty-one patients with metastatic renal carcinoma were referred to our institute from October 1987 until August 1991; 129 were treated with Interleukin 2 with or without Interferon alpha in three successive protocols. Fifty-two patients were not treated with immunotherapy due to the exclusion criteria of the protocols. Sixty-four patients with the same disease who had been referred to our institute before the initiation of this programme (1982, 1987) were also analysed as a control group. The main characteristics of the three different cohorts of patients were analysed and compared with univariate statistical tests; the median survival of the patients was calculated and compared.

The referral rate increased from 13 a year to 45 a year while the IL2 trials were being conducted.

Patients treated with cytokines have a median survival of 18 months after occurrence of metastases, compared to 6 and 8 months, respectively, in excluded patients and the control group. This parameter is of 15 months when the 181 patients, treated with cytokines or not, are considered. The survival of treated *vs* excluded patients is significantly different ($P < 10^{-6}$); so is the survival of the 181 patients recently included when compared to the historical group ($P$:$10^{-5}$). When the 181 recent patients are compared to the historical control group, a number of differences appear in their characteristics, which prevent us from drawing any conclusion about the role of immunotherapy in the improvement of survival observed.

This study clearly evidences the selection of the patients receiving immunotherapy and the modification in referrals of a disease induced by a new available therapy. This emphasises the need for prospective studies in this setting.

Most conventional systemic therapies have little or no activity in advanced renal carcinoma (Droz *et al*., 1988; Yagoda *et al*., 1989). Immunotherapy with Interferon or Interleukin-2 (IL2), used alone or in combination, has proved to be active in this disease, leading to response rates ranging from 15 to 30% (Quesada *et al*., 1983; Krown, 1987; Bergerat *et al*., 1988; Rosenberg *et al*., 1987; 1989*a*,*b*; West *et al*., 1987; Négrier *et al*., 1989; 1992; Atzpodien *et al*., 1990). However, the toxicity induced by these cytokines is of great concern and raises the question of the justification of this treatment modality (Moertel, 1986). According to previous studies, the median survival time is of 6 to 12 months in patients with metastatic renal carcinoma (Maldazys *et al*., 1986; Ritchie *et al*., 1987; Forges de *et al*., 1988; Philip *et al*., 1989). Very few consistent data are available yet in the literature concerning the impact of immunotherapy on survival (Palmer *et al*., 1992*a*). The present study was undertaken first to analyse the whole cohort of patients accrued during almost 4 years. We studied their characteristics, prognostic factors as well as their survival, considering whether they received cytokine therapy or not. Secondly, we analyse the differences of this cohort when compared to our historical group.

### Patients and methods

### *Patients and treatments*

Three different groups of patients have been defined in this study; their main characteristics are detailed in Table I. All therapeutic protocols using unregistered drugs were conducted after local ethical committee acception and patient informed consent.

Correspondence: T. Philip, Centre L. Bérard, 28 rue Laënnec, 69373 Lyon Cédex 08, France.
Received 19 March 1993; and in revised form 12 July 1993.

Almost all patients (at least those included in phase II trials i.e. 162/181) accrued after initiation of our immunotherapy programme, underwent the same investigation procedures to assess eligibility and to screen tumour localisations. Cranial, thoracic, abdominal and pelvic CT scan, bone scintigraphy and blood controls of major organ functions were performed.

Patients receiving cytotoxic regimens were reevaluated with at least thoracic and abdominal CT scans combined with other procedures if necessary.

### *Included patients*

One hundred and twenty-nine patients were treated with cytokines within the immunotherapy programme. Sixty of these patients received IL2 as a continuous infusion (Eurocetus BV Amsterdam, The Netherlands) at $18 \times 10^6$ IU/m$^2$/day, according to the schedule previously reported by West *et al*. (1987); LAK cells were associated with IL2 infusion in 22 patients. Thirty-four patients received intravenous IL2 combined with Interferon alpha (IFN) (Schering Plough, Paris, France) according to the following schedule: one subcutaneous injection of IFN at $20 \times 10^6$/IU per day for 5 days then, after a 2-day rest, IL2 as bolus doses at $24 \times 10^6$ IU/m$^2$ q 3/day combined with intravenous IFN at $5 \times 10^6$ IU/m$^2$ q 3/day for 5 days. After a 6-day break, during which cytaphereses were performed in order to develop LAK cells, the same intravenous combined schedule was carried on for five additional days along with the LAK cell reinjection. Twenty-five patients received a combination of subcutaneous IL2 and IFN according to the schedule of Atzpodien *et al*. (1990), previously described. Ten patients, who had received IFN therapy before being referred to our institute were treated with a combined therapy of IL2 and Tumor Necrosis Factor (TNF), as previously reported (Négrier *et al*., 1992).

**Table I** Characteristics .of the three groups of patients

| | *Control* | *Excluded* | *Included* |
|---|---|---|---|
| No. of patients | 64 | 52 | 129 |
| Age median (range) | 59 (25–80) | (59 (36–75) | 58 (24–77) |
| *Gender (%)* | | | |
| Males | 44 (69) | 40 (77) | 94 (73) |
| Females | 20 (31) | 12 (23) | 35 (27) |
| No. patients with initial metastases (%) | 33 (52) | 36 (69) | 71 (55) |
| Median time between diagnosis and metastases (months) (If no initial metastasis) | 23 | 19 | 21 |
| *Sites of disease (%)* | | | |
| Lung | 41 (64) | 42 (81) | 110 (85) |
| Bone | 40 (63) | 31 (59) | 54 (42) |
| Liver | 4 (6) | 9 (17) | 24 (19) |
| Brain | 5 (8) | 11 (21) | 6 (5) |
| Other sites | 40 (62) | 25 (48) | 61 (47) |
| Local relapse | 9 (14) | 5 (10) | 17 (13) |
| *No. of metastatic sites (%)* | | | |
| ⩽1 | 23 (36) | 8 (15) | 34 (26) |
| 2 | 31 (48) | 23 (44) | 58 (45) |
| 3 | 9 (14) | 15 (28) | 33 (26) |
| ⩾4 | 1 (1) | 6 (11) | 4 (3) |
| Prior nephrectomy (%) | 46 (71) | 43 (82) | 113 (88) |
| Adjuvant therapy (%) | 12 (19) | 5 (10) | 8 (6) |
| *No. of systemic therapeutic lines (%)* | | | |
| 0 | 31 (48) | 21 (40) | 0 (0) |
| 1 | 23 (36) | 22 (42) | 60 (46) |
| 2 | 10 (16) | 8 (15) | 44 (34) |
| ⩾3 | 0 (0) | 1 (2) | 25 (20) |
| Radiotherapy (%) | 44 (69) | 27 (52) | 53 (41) |

*Excluded patients*

Between October 1987 and August 1991, 52 (29%) patients were excluded from immunotherapy because of exclusion criteria defined in these protocols. Notably, brain metastases were considered as an exclusion criterion until April 1990. Patients who were referred after this date, with previously treated brain metastases (surgery or radiotherapy) without evidence of evolutive lesions in brain, received IL2 as continuous infusion. The main reasons of exclusion were respectively: ECOG performance status score under 1 (18), brain metastases (11), hypercalcemia (5), absence of measurable tumour lesions (4), refusal (2), ongoing steroid therapy (2), acute complication due to tumour progression (2), cardiac failure (1), chronic renal failure (1). We endeavour to give these patients chemotherapy regimens as phase II therapeutic trials. Eighteen (35%) were treated with intravenous fotemustine (Servier, Paris, France) (at least three injections separated by 2 week rest); 15 (29%) received continuous infusion of FUDR i.e. 5-fluoro-desoxyuracile (Roche, Paris, France) via an electronic portable pump. Nineteen (36%) received only palliative support including local radiotherapy.

*Control patients*

Sixty-four patients with metastatic renal cell cancer were referred to our institute between 1982 and 1987. Their main characteristics are detailed in Table I. Thirty-three patients (51%) received systemic therapy (e.g. hormone therapy in 21 and various regimens of chemotherapy in 20) as treatment of metastatic disease. Five patients only were included in phase II trials. Sixty-eight percent (44/64) patients received radiotherapy mainly for pain control and, in 27 (42%) patients this was the only anticancer treatment performed.

*Statistical analysis*

The parameters taken into account in the present study, when available, were those considered in previous reports on prognostic factors in metastatic renal carcinoma (Forges de *et al*., 1988; Elson *et al*., 1988; Palmer *et al*., 1992*b*). Comparisons between the different cohorts of patients were performed using the chi-square test for categorical data.

Survival data were evaluated from the date of initial diagnosis or of first metastases until the date of death or of last contact, when the patient is still alive. Survival curves were calculated using the Kaplan Meïer method, and univariate comparisons between curves were done using the log-rank test; results are expressed as relative risk (RR).

A multivariate prognosis analysis for survival could not be performed since some important parameters, i.e. performance status and weight loss, were not available in the control group.

### Results

*Response to treatment*

No significant tumour regression was observed in the excluded patients but five stabilisation of disease for at least 3 months were noted in the patients treated by chemotherapy.

In the 129 treated patients, five complete responses and 16 partial responses (response rate 16%) were achieved and the details concerning the responding patients are summarised in Table II. All responders but one had previously undergone nephrectomy, the median number of metastatic sites was 2 and the median duration of response was 8 months. Stabilisation of the disease was achieved in 42 patients.

No objective tumour regression had been observed within the historical control group, but ten patients had been considered as stabilised during at least 3 months.

*Comparison between the different groups of patients*

The main features of the three different cohorts of patients were compared in an univariate analysis. Table III shows the results of the comparisons between patients included and patients excluded from the immunotherapy protocols.

Two parameters that evidence significant differences had been previously defined as two exclusion criteria of immunotherapy protocols. Indeed, patients with brain metastases were not eligible for the first two protocols. Patients with an ECOG performance status beneath 1 were ineligible and this last parameter is dramatically different in the two cohorts (*P* value $<10^{-5}$). Three other parameters are also significantly different, the number of patients with bone metastases is higher within the excluded group, as well as the number of patients with previous therapy (e.g. chemotherapy or radio-

Table II Characteristics of responders according to immunotherapy protocols

| Patients and regimens | Gender | Age | Prior nephrectomy | No. of metastatic sites | Response and duration (months) |
|---|---|---|---|---|---|
| IL2 alone $n = 60$ | | | | | |
| 1 | M | 36 | + | 3 | PR (8) |
| 2 | M | 51 | + | 2 | PR (4) |
| 3 | M | 50 | + | 2 | PR (21) |
| 4 | M | 55 | + | 3 | PR (8) |
| 5 | M | 69 | + | 3 | PR (4) |
| 6 | M | 75 | + | 2 | CR (24+) |
| 7 | M | 65 | + | 1 | CR (22+) |
| 8 | M | 67 | + | 1 | PR (8+) |
| 9 | M | 34 | + | 1 | PR (9+) |
| IL2 + IFN HD $n = 34$ | | | | | |
| 1 | M | 50 | + | 3 | PR (6) |
| 2 | M | 63 | + | 2 | PR (5) |
| 3 | M | 63 | + | 1 | CR (12) |
| 4 | M | 58 | + | 2 | CR (23+) |
| 5 | F | 60 | + | 2 | PR (4) |
| 6 | M | 49 | + | 2 | PR (4) |
| 7 | M | 56 | – | 2 | PR (CR*) (18+) |
| 8 | M | 51 | + | 1 | PR (12+) |
| IL2+IFN LD $n = 25$ | | | | | |
| 1 | M | 59 | + | 1 | PR (4) |
| 2 | M | 65 | + | 2 | PR (17+) |
| 3 | M | 55 | + | 1 | CR (13) |
| IL2+TNF $n = 10$ | | | | | |
| 1 | M | 60 | + | 2 | PR (4) |

*CR post surgery on renal tumour. HD: high doses; LD: low doses; PR: partial response; CR: complete response.

Table III Comparison of the treated *vs* excluded patients

| Parameters | Excluded (%) | Treated (%) | P-value (chi-square test) |
|---|---|---|---|
| Gender (males) | 77 | 73 | 0.573 |
| No nephrectomy | 17 | 12 | 0.387 |
| No adjuvant therapy | 92 | 94 | 0.691 |
| Local relapse | 10 | 13 | 0.507 |
| No. metastatic sites >2 | 40 | 29 | 0.127T |
| Brain metastases (yes) | 21 | 5 | 0.001[a] |
| Lung metastases (yes) | 81 | 85 | 0.455 |
| Liver metastases (yes) | 17 | 19 | 0.838 |
| Bone metastases (yes) | 59 | 42 | 0.030[a] |
| Other metastatic sites (yes) | 49 | 47 | 0.923 |
| Patients >65 years at metastases | 23 | 20 | 0.662 |
| Initial metastases | 69 | 55 | 0.079T |
| *Previous therapy* | 37 | 19 | 0.008[a] |
| chemotherapy | 14 | 1 | 0.001[a] |
| hormonotherapy | 2 | 2 | 0.868 |
| radiotherapy | 31 | 9 | 0.0001 |
| Previous stabilisation of disease ⩾4 months | 20 | 31 | 0.141 |
| Weight loss ⩾10% | 48 | 26 | 0.003[a] |
| Performance status ECOG ⩾2 | 40 | 11 | $<10^{-5}$ |

[a]Significant $P$ value. T = trend.

therapy) and the number of patients with a weight loss of at least 10% of their basal weight.

Two additional parameters, thought their $P$ value does not reach the significant threshold, indicate a difference in these two populations, i.e. the number of patients with more than two tumour sites and the number of patients with initial metastases.

In Table IV, we compared the historical control group *vs* the overall cohort of the later patients eligible or not for immunotherapy. Despite the lack of information for two major prognostic factors (i.e. weight loss and performance status), a number of parameters are different in these two populations. Nephrectomy, lack of adjuvant therapy, lung and liver metastases are less frequent in the control group as well as the number of treatments received. In contrast, the number of patients above 65 at occurrence of metastases is higher in the control group, and the use of radiotherapy is more frequent.

In addition, we see a difference in the accrual rate: 13 (64/5 years) patients per year in the control group *vs* 45 (181/4 years) in the latter group.

*Survival analysis*

If we consider the survival from the occurrence of metastases, the median time of follow-up is 37 months (range: 18–71) in excluded patiens *vs* 43 months (range: 19–123) in treated patients. It was 103 months (range 59–150) in the control group.

According to the survival curves shown in Figure 1, the median survival time of the control group, of the excluded group and of the treated group are respectively 8, 6 and 18 months. The difference between the excluded group and immunotherapy treated patients is significant (RR: 2.28; $P<10^{-6}$) whereas it is not different between the excluded and the control group (RR: 1.01; $P = 0.94$). As shown in Figure 2, excluded patients were pooled together with treated patients and the median survival time was then of 15 months. The difference between this population and the control group remains significant (RR: 1.85; $P = 10^{-5}$).

When the survival from the date of initial diagnosis is considered, the median times of follow-up are 43, 37 and 103 months respectively in the immunotherapy, the excluded and the control groups, with a respective median survival of 24, 9 and 13 months (Figure 3). The survival difference between the excluded group and the immunotherapy treated patients is significant (RR: 1.88; $P = 10^{-4}$). Figure 4 shows the survival from initial diagnosis of both groups brought together. The median survival is then of 20 months and remains significantly different from that of the control group (RR: 1.37; $P = 0.02$).

### Discussion

Even if rigorous criteria, such as WHO criteria for tumour evaluation are used, the efficacy of immunotherapy with Interleukin 2 in solid tumours has been judged until now mainly on the response rates of non randomised phase II studies (Rosenberg *et al.*, 1987; 1989*b*; Négrier *et al.*, 1989; Atzpodien *et al.*, 1990). IL2 induces objective responses in

**Table IV** Comparison of control patients *vs* prospective cohort

| *Parameters* | *Control (%)* | *Prospective cohort (%)* | *P value (chi-square test)* |
|---|---|---|---|
| Gender (males) | 68 | 74 | 0.339 |
| No nephrectomy | 28 | 13 | 0.010[a] |
| No adjuvant therapy | 81 | 93 | 0.005[a] |
| Local relapse | 20 | 12 | 0.109 |
| No. of metastatic sites >2 | 16 | 32 | 0.01 |
| Brain metastases (yes) | 5 | 9 | 0.237 |
| Lung metastases (yes) | 64 | 84 | 0.001 |
| Liver metastases (yes) | 6 | 18 | 0.021[a] |
| Bone metastases (yes) | 56 | 47 | 0.201 |
| Other metastatic sites (yes) | 39 | 47 | 0.243 |
| No. patients >65 years at metastases | 36 | 21 | 0.017[a] |
| Initial metastases | 51 | 59 | 0.294 |
| No. of systemic lines ⩾2 (immunotherapy included) | 16 | 43 | 0.0007[a] |
| Radiotherapy | 69 | 44 | 0.001[a] |

[a]Significant *P* value.

**Figure 1** Survival of IL2 treated patients *vs* excluded *vs* control group from the occurrence of metastases. --- IL2 $n = 129$; ----- Excluded $n = 52$; ——— Control group $n = 64$.

**Figure 2** Survival of the IL2 patients together with excluded patients *vs* control group from the occurrence of metastases. ------ IL2 + excluded $n = 181$; ——— Control group $n = 64$. The survival rate at 18 months was: – IL2 + excluded: 43% (confidence interval ± 8%). – Control: 19% (confidence interval ± 10%).

only a minority of patients whereas its related toxicity concerns all of them (Siegel *et al.*, 1991). We thus attempted to analyse the survival and the characterstics of patients with metastatic renal carcinoma who were referred to our institute, whether they received immunotherapy or not.

The most conspicuous conclusion that can be drawn from this study is that patients eligible to receive immunotherapy are selected. Indeed, 29% of our patients were excluded from IL2 protocols. This group, as shown by the analysis of their characteristics and prognostic factors as well as their sur-

**Figure 3** Survival of IL2 treated patients *vs* excluded *vs* control group from initial diagnosis. --- IL2 $n = 129$; ------ Excluded $n = 52$; ——— Control group $n = 64$.

**Figure 4** Survival of IL2 treated patients together with excluded patients *vs* control group from initial diagnosis. ------- IL2 + excluded $n = 181$; ——— Control group $n = 64$. The survival rate at 5 years (60 months) was: - IL2 + excluded: 26% (confidence interval ± 8%). - Control: 15% (confidence interval ± 10%).

vival, represents a bad prognosis group with a limited survival (e.g. median survival of 6 months after occurrence of metastases).

These selection biases lead to an artefactual improvement of the survival of treated patients when compared to the overall survival of the population suffering from the same disease.

Selection of patients is not restricted to this kind of therapy, but is a common problem in the evaluation of treatments, specially in oncology (Peto *et al.*, 1976; 1977).

Very few authors, however, mention the exclusion rate of their clinical trials, but they draw conclusions from a selected group that are often taken into account for the whole population. We thus think that the reports of phase II trials in oncology should indicate the concomittent exclusion rate.

If we consider patients as a whole, whether treated with immunotherapy or not, the median overall survival is 16 months after occurrence of metastases. Since the literature reports no series with a median survival time over 1 year, we attempted to appreciate the factors that could be responsible for this improvement. With this aim, we analysed our historical control group, i.e. patients referred to our institute during the 5 years preceding initiation of the immunotherapy programme, in which the median survival is of 8 months. Such a median survival of 8 months has also been reported in another cohort of French patients with the same disease receiving different regimens of chemotherapy as phase II trials (Forges de *et al.*, 1988). Our results were weakened by the lack of two important prognostic factors which were not available in the retrospective cohort; i.e., weight loss and performance status.

Performance status appeared in three previous studies as a very powerful prognostic factor (de Forges, 1988; Elson 1988; Palmer, 1992*b*). Therefore, matching the patients on other available characteristics was judged as not suitable.

Despite the fact that these two prognostic factors, i.e. weightloss and performance status, were not available in the retrospective series, the analysis of this group and the comparison with the latter group show a number of differences that correspond to selection biases. First of all, the accrual rate is quite different, i.e. 13 patients/year *vs* 45, which proves that referrals of this disease are very much stimulated by available therapy. Notably, drawn from the incidence rate of this disease and the number of inhabitants in the surrounding area, we estimated that approximately half the patients suffering from metastatic renal cancer were currently referred to our institution.

In addition, three parameters that are significantly different could partially explain the difference in survival, independently from the receiving of immunotherapy. Though median ages are identical, there are more patients over 65 with occurrence of metastases and, in addition, a greater number of patients who did not undergo nephrectomy within the control group. Control patients received fewer systemic therapeutic lines than the immunotherapy treated patients, and this parameter might influence their survival as well.

Conversely, we note that the number of control patients with more than two tumour sites is reduced, as well as the number of patients with lung and liver metastases. This difference in the number of metastatic sites would indicate that, despite a larger tumour burden, the survival of patients treated recently has been significantly improved. We must, however, point out that this last parameter should be considered with caution since the detection of metastatic sites was very different in these two cohorts. In fact, in the control group, metastatic lesions were merely detected when they became symptomatic, whereas a complete initial screening was performed in almost all the patients belonging to the second cohort, i.e. at least all patients in phase II trials (162/181: 89%). For this reason, the bulk of the disease was probably underestimated in our historical group.

As a result of selection bias and lead time bias, the two populations are obviously different. For this reason, it is not possible to appreciate the role of immunotherapy in the improved survival observed in recent patients. Notably, the starting point of survival evaluation, i.e. initial diagnosis *vs* first metastases, does not influence the results of our study, but reduced the difference in survival. Indeed, the survival of the control group appeared somewhat more favourable when analysed from initial diagnosis, whereas the shape of the curve is not modified in the prospective group. We hypothetised that the diagnosis of metastases has been done later in the course of the disease in the control than in the prospective group.

This analysis emphasises the impact of a new specific therapeutic programme and the management or follow-up modifications induced by the close evaluation of controlled trials (Osband *et al*., 1990).

As a consequence, survival evaluation, especially for immunotherapy in metastatic renal cancer, is difficult. The most logical and objective way to appreciate the real impact of immunotherapy on survival would be to compare, within a prospective randomised trial, a treated group *vs* a placebo group. This situation, however, is ethically unfair since we know that immunotherapy can bring, although in only some patients, durable complete remissions.

Therefore, we now have to appreciate the response rate and survival of patients treated with different cytokine schedules in prospective multicentric trials and to try and evidence the predictive factors of response to therapy. Such a study is presently ongoing in France in metastatic renal carcinoma.

In summary, this study shows that the administration of immunotherapy with cytokines results in a selection of patients with the exclusion of a poor survival group. Therefore, the exclusion rate should also be investigated and reported in phase II trials in this setting. It also demonstrates the impact of a new therapeutic modality on referrals of a disease. The survival of patients with metastatic renal carcinoma treated with immunotherapy remains to be investigated specifically in a prospective and comparative setting.

This work was supported by the Ligue Nationale contre le Cancer.

### References

ATZPODIEN, J., KORFER, A., FRANKS, C.R., POLIWODA, H. & KIRCHNER, H. (1990). Home therapy with recombinant interleukin-2 and interferon Alpha in advanced malignancies. *Lancet*, **335**, 1509–1512.

BERGERAT, J.P., HERBRECHT, R., DUFOUR, P., JACQMIN, D., BOLLACK, C., PREVOT, G., BAILLY, G., GARIS DE, S., JURASCHEK, F. & OBERLING, F. (1988). Combination of recombinant alpha 2A interferon and vinblastine in advanced renal cell cancer. *Cancer*, **62**, 2320–2324.

DROZ, J.P., THEODORE, C., GHOSN, M., LUPERA, H., PIOT, G., FORGES DE, A., KLINK, M., RONESSE, J. & AMIEL, J.L. (1988). Twelve years experience with chemotherapy in adult metastatic renal cell carcinoma at the Institut Gustave Roussy. *Sem. Oncol.*, **4**, 97–99.

ELSON, P.J., WITTER, S. & TRUMP, D.L. (1988). Prognostic factors for survival in patients with recurrent or metastatic renal cell carcinoma. *Cancer Res.*, **48**, 7310–7313.

FORGES DE, A., REY, A., KLINK, M., GHOSN, M., KRAMAR, A. & DROZ, J.P. (1988). Prognostic factors for adult metastatic renal carcinoma: a multivariate analysis. *Sem. Surg. Oncol.*, **4**, 149–154.

KROWN, S.E. (1987). Interferon treatment of renal cell carcinoma. Current status and future prospects. *Cancer*, **59**, 647–651.

MALDAZYS, J.D. & DE KERNION, J.B. (1986). Prognostic factors in renal cell carcinoma. *J. Urol.*, **136**, 376–379.

MOERTEL, C.G. (1986). On lymphokines, cytokines and breakthroughs. *J. Am. Med. Assoc.*, **256**, 3141–3145.

NÉGRIER, S., PHILIP, T., STOTER, G., FOSSA, S.D., JANSSEN, S., IACONE, A., CLETON, F.S., EREMIN, O., ISRAEL, L., JASMIN, C., RUGARLI, C., MAAS, H.V.D., THATCHER, N., SYMANN, M., BARTSCH, H.H., BERGMANN, L., BIJMAN, J.T., PALMER, P.A. & FRANKS, C.R. (1989). Interleukin 2 with or without LAK cells in metastatic renal cell carcinoma: a report of a European multicentric study. *Eur. J. Cancer Clin. Oncol.*, **251**, S19–S28.

NÉGRIER, M.S., POURREAU, C.N., PALMER, A., RANCHÈRE, J.Y., MERCATELLO, A., VIENS, P., BLAISE, D., JASMIN, C., MISSET, J.L., FRANKS, C.R., MARANINCHI, D. & PHILIP, T. (1992). Phase I trial of recombinant Interleukin-2 followed by recombinant tumor necrosis factor in patients with metastatic cancer. *J. Immunotherapy*, **11**, 93–102.

NÉGRIER, S., MERCATELLO, A., BRET, M., THIESSE, P., BLAY, J.Y., CORONEL, B., MERROUCHE, Y., OSKAM, R., FRANKS, C.R., CLAVEL, M., MOSKOVTCHENKO, J.F. & PHILIP, T. (1992). Intravenous Interleukin-2 in patients over 65 with metastatic renal carcinoma. *Br. J. Cancer*, **65**, 723–726.

OSBAND, M.E. & ROOS, S. (1990). Problems in the investigational study and clinical uses of cancer immunotherapy. *Immunol. Today*, **11**, 193–195.

PALMER, P.A., VINKE, J., EVERS, P., POURREAU, C., OSKAM, R., ROEST, G., VLEMS, F., BECKER, L., LORIAUX, E. & FRANKS, C.R. (1992*a*). Continuous infusion of recombinant interleukin with or without autologous lymphokine activated killer cells for the treatment of advanced renal cell carcinoma. *Eur. J. Cancer*, **28**, 1038–1044.

PALMER, P.A., VINKE, J., PHILIP, T., NÉGRIER, S., ATZPODIEN, J., KIRCHNER, H., OSKAM, R. & FRANKS, C.R. (1992*b*). Prognostic factors for survival in patients with advanced renal cell carcinoma treated with recombinant interleukin-2. *Ann. Oncol.*, **3**, 475–480.

PETO, R., PIKE, M.C., ARMITAGE, J., BRESLOW, N.E., COX, D.R., HOWARD, S.V., MANTEL, N., MCPHERSON, K., PETO, J. & SMITH, P.G. (1976). Design and analysis of randomized clinical trials requiring prolonged observation of each patient. I. Introduction and design. *Br. J. Cancer*, **34**, 585–612.

PETO, R., PIKE, M.C., ARMITAGE, J., BRESLOW, N.E., COX, D.R., HOWARD, S.V., MANTEL, N., MCPHERSON, K., PETO, J. & SMITH, P.G. (1977). Design and analysis of randomized clinical trials requiring prolonged observation of each patient. II. Analysis and examples. *Br. J. Cancer*, **35**, 1–39.

PHILIP, T., MERCATELLO, A., NÉGRIER, S., PHILIP, I., REBATTU, P., KAEMMERLEN, P., GASPARD, M., TOGNET, E., COMBARET, V., BIJMAN, J.T., FRANKS, C.R., CHAUVIN, F., MOSKOVTCHENKO, J.F., FAVROT, M. & CLAVEL, M. (1989). Interleukin-2 with or without LAK cells in metastatic renal cell carcinoma: the Lyon first year experience on 20 patients. *Cancer Treat Rev.*, **16**, 91–104.

QUESADA, J.R., SWANSON, D.A., TRINDADE, A. & GUTTERMAN, J.U. (1983). Renal cell carcinoma: antitumour effects of leukocyte interferon. *Cancer Res.*, **43**, 940–947.

RITCHIE, A.W.S. & DE KERNION, J. (1987). The natural history and clinical features of renal carcincoma. *Sem. Nephrol.*, **7**, 131–139.

ROSENBERG, S.A., LOTZE, M.T., MUUL, L.M., CHANG, A.E., AVIS, F.P., LEITMAN, S., LINEHAN, W.M., ROBERTSON, C.N., LEE, R.E., RUBIN, J.T., SEIPP, C.A., SIMPSON, C.G. & WHITE, D.E. (1987). A progress report on the treatment of 157 patients with advanced cancer using lymphokine activated killer cells and interleukin 2 or high dose interleukin 2 alone. *N. Engl. J. Med.*, **313**, 889–897.

ROSENBERG, S.A., LOTZE, M.T., YANG, J.C., LINEHAN, M., SEIPP, C., CALABRO, S., KARP, S.E., SHERRY, R.M., STEINBERG, S. & WHITE, D.E. (1989*a*). Combination therapy with Interleukin 2 and alpha-Interferon for the treatment of patients with advanced cancer. *J. Clin. Oncol.*, **7**, 863–874.

ROSENBERG, S.A., LOTZE, M.T., YANG, J.C., AEBERSOLD, P.M., LINEHAN, W.M., SEIPP, C.A. & WHITE, D.E. (1989*b*). Experience with the use of high dose interleukin 2 in the treatment of 652 patients with cancer. *Ann. Surg.*, **210**, 474–485.

SIEGEL, J.P. & PURI, R.K. (1991). Interleukin-2 toxicity. *J. Clin. Oncol.*, **9**, 694–704.

WEST, W.H., TAUER, K.W., YANELLI, J.R., MARSHALL, G.D., ORR, D.W., THURMAN, G.B. & OLDHAM, R.K. (1987). Constant infusion recombinant interleukin-2 in adoptive immunotherapy of advanced cancer. *N. Engl. J. Med.*, **316**, 898–905.

YAGODA, A. (1989). Chemotherapy of renal cell carcinoma. *Sem. Urol.*, **7**, 199–206.